# Ancient genes can be served as pan‐cancer diagnostic and prognostic biomarkers

**DOI:** 10.1111/jcmm.15347

**Published:** 2020-05-05

**Authors:** Xiangwen Ji, Qinghua Cui

**Affiliations:** ^1^ Department of Biomedical Informatics Department of Physiology and Pathophysiology Center for Noncoding RNA Medicine School of Basic Medical Sciences Peking University Beijing China; ^2^ Co., Ltd of JeanMoon Beijing China

**Keywords:** ancient genes, diagnosis, pan‐cancer, progenitorness score, prognosis

## Abstract

One important challenge for cancer is efficient biomarkers monitoring its formation and developments remain greatly limited. Although the accumulated big omics data provide great opportunities to the above purpose, the biomarkers identified by the data‐driven strategy often do not work well in new datasets, which is one of the main bottlenecks limiting their utilities. Given that atavistic phenotype is generally observed in cancer cells, we have been suggested that the activity of progenitor genes in tumour could serve as an efficient cancer biomarker. For doing so, we first curated 77 progenitor genes and then proposed a quantitative score to evaluate cancer progenitorness. After applying progenitorness score to ~ 22 000 samples, 33 types of cancers from 81 datasets, this method generally performs well in the diagnosis, prognosis and therapy monitoring of cancers. This study proposed a potential pan‐cancer biomarker and revealed a significant role of atavism in the formation and development of cancers.

## 1 INTRODUCTION

With the progress of medical technology and the extension of human life expectancy, non‐communicable diseases have become the main cause of threats to human life, especially the cancers.^1^ It is estimated that there were more than 18.1 million new cancer cases and 9.6 million deaths worldwide in 2018.^1^, ^2^ Precise biomarkers are believed to be of great important for cancer diagnosis and therapy but currently remain great limited. Recently, the accumulated big omics‐data provides great opportunities for exploring cancer biomarkers, however, the biomarkers found by this data‐driven strategy often do not work well on new datasets.^3^ Currently, although some biomarkers have been discovered and applied in the diagnosis, treatment and prognosis of cancers,^4^, ^5^, ^6^ due to the heterogeneity and diversity of diseases, the deficiency of efficient biomarkers represents one of the main bottlenecks limiting cancer medicine.^7^, ^8^ In order to diagnose and treat effectively, extend the survival time and improve the prognosis, there is an urgent need for universal and effective diagnosis and prognostic evaluation biomarkers.

Atavism is considered as a reappearance of lost progenitor phenotype in genetics.^9^ In the last decade, the hypothesis that cancer is an atavistic condition was proposed, which holds that the genes related to cell cooperation that evolved into multicellular organisms about 1 billion years ago match the genes that cause cancer. This 'unlock of ancient toolkit' leads to the occurrence of cancer.^10^ Cancer cells are moulded into protozoan‐like organisms whose original specific functions and characteristics disappear and become purely for survival.^11^ Another study pointed out that in the process of drug resistance of tumours, in addition to the accumulation of somatic mutations, which is the traditional view of the occurrence of cancer, there is also the regulation of some non‐mutated genes, which is very ancient and conservative and may be the basis of life.^12^ Thus, atavistic model can be combined with somatic selection model as a new explanation for the occurrence and development of cancer.^13^

Given the above observation, we have been suggested that progenitor genes might serve as biomarkers for cancer. To confirm this hypothesis, we first curated 77 progenitor genes according to the phyletic age and the essentiality of genes from the database of Online Gene Essentiality (OGEE).^14^, ^15^ Then we proposed a score to quantify the progenitorness of a sample using its expression profile data. By applying progenitorness score to 33 types of cancers of 81 datasets from the Cancer Genome Atlas (TCGA), the Chinese Glioma Genome Atlas (CGGA), the Gene Expression Omnibus (GEO) and the Integrative Molecular Database of Hepatocellular Carcinoma (HCCDB)^16^ databases, we showed that the proposed progenitorness score work efficiently for the diagnosis, prognosis, grading and monitoring therapy of various cancers.

## 2 MATERIALS AND METHODS

### 2.1 Curation of progenitor genes and single‐sample gene set enrichment analysis

Given that this study is based on the atavism hypothesis of cancer, here we aim to investigate whether ancient progenitor genes can be used as cancer biomarkers. The database of Online Gene Essentiality (OGEE, http://ogee.medgenius.info/browse/) defines the phyletic age and the essentiality of genes. Six different phyletic ages for the genes were defined from ancient to present: cellular organisms, Eukaryota, Fungi/Metazoa group, Metazoa, Chordata and Mammalia. We define the progenitor genes as essential genes whose phyletic ages are cellular organisms or Eukaryota. As a result, we curated a total of 77 progenitor genes (File S1). To calculate the progenitorness score, we perform single‐sample gene set enrichment analysis^17^ (ssGSEA) of gene expression profiles in the progenitor gene set by python (v3.6.8) package gseapy (v0.9.16), which is a python wrapper for GSEA and ssGSEA.

### 2.2 Data collection and pre‐processing

TCGA RNA sequencing (RNAseq) data in fragments per kilobase of transcript per million mapped reads (FPKM) and clinical information were downloaded from GDC data portal (https://portal.gdc.cancer.gov/). Histology type and WHO grade of TCGA lower grade glioma (LGG) and glioblastoma multiforme (GBM) were obtained from the study by Ceccarelli et al^18^ CGGA (http://www.cgga.org.cn/) contains gene expression and clinical data of more than 1000 patients with glioma, which are separated into one microarray and two RNAseq batches, giving researchers an opportunity to delve deeper into glioma. HCCDB^16^ (http://lifeome.net/database/hccdb/home.html) provides 15 public hepatocellular carcinoma (HCC) gene expression matrices from TCGA, the International Cancer Genome Consortium (ICGC) and GEO datasets, which were processed in a unified process. HCC proteome data were obtained from Gao et al^19^ In addition, microarray gene expression profiling data and RNAseq data were collected from GEO database (https://www.ncbi.nlm.nih.gov/gds/). Cancer Cell Line Encyclopedia^20^ (CCLE, https://portals.broadinstitute.org/ccle/) provides RNAseq data for thousands of cancer cell lines. Gene expression data were structured with gene symbols as row names, sample ids as column names, duplicate gene symbols were dropped except their max value. Drug sensitivity data are available from Cancer Therapeutics Response Portal^21^ (CTRP, http://portals.broadinstitute.org/ctrp/)and Genomics of Drug Sensitivity in Cancer^22^ (GDSC, https://www.cancerrxgene.org/).

### 2.3 Statistical Analysis

All statistical significances were calculated by R (v3.5.2). Cox proportional hazards regression and Kaplan‐Meier (K‐M) curves were processed by R package survival (v3.1‐7) and survminer (v0.4.6). Log‐rank test was used to evaluate the difference between two K‐M curves. Receiver operating characteristic (ROC) curve and area under ROC curve (AUROC) were performed by R package pROC^23^ (v1.15.3). Significance of difference between two groups of continuous variables was calculated by two‐side Wilcoxon rank‐sum test. Differences of paired samples were evaluated by Wilcoxon signed‐rank test. Spearman's tests were used to estimate correlation between two continuous variables. The *P* values of Spearman's test were adjusted using R package fdrtool (v1.2.15).

## 3 RESULTS

### 3.1 Progenitorness score distinguishes tumours from normal samples

**Figure 1:**
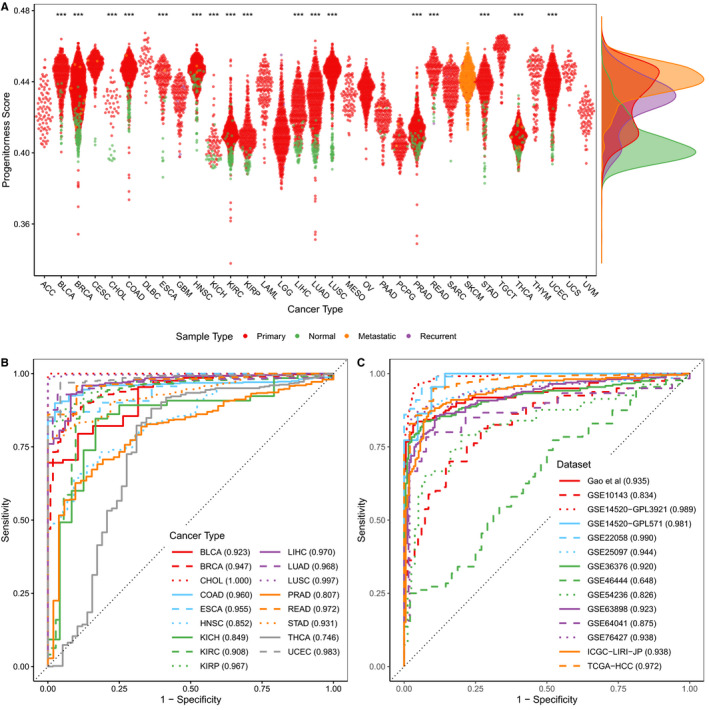
Progenitorness score distinguishes tumours from normal samples. A, Distribution of progenitorness score in different cancer types and sample types in TCGA. Significances of difference between primary tumours and normal tissues were analysed by two‐side Wilcoxon rank‐sum test. ***P < 0.001. B, ROC curves of progenitorness scores discriminating primary tumours from normal tissues in TCGA. (C) ROC curves of progenitorness scores discriminating primary tumours from normal tissues in HCCDB. The area under ROC curves are shown in parentheses. The cancer type abbreviations of TCGA is in https://gdc.cancer.gov/resources‐tcga‐users/tcga‐code‐tables/tcga‐study‐abbreviations

Firstly, we investigated whether the proposed progenitorness score is able to distinguish tumour samples from normal samples. As expected, primary tumours showed significantly higher progenitorness scores than normal tissues for all 17 types of cancers in the TCGA database (Figure 1A). Moreover, progenitorness score showed a good prediction performance in distinguishing tumours from normal samples (Figure 1B). We obtained similarity results in datasets from GEO and HCCDB (Figure 1C, Figure S1, S2). We noted that progenitorness score did not work well on only one dataset (GSE46444), which could be resulted from the fact that the samples of this dataset were formalin‐fixed paraffin‐embedded (AS‐FFPE). In addition, the GSE25097 dataset has samples of cirrhotic liver. As expected, the progenitorness scores of cirrhotic livers are between those from the cancer samples and those from the adjacent samples (Figure S2E, I).

### 3.2 Progenitorness score predicts the survival of cancer patients

**Figure 2:**
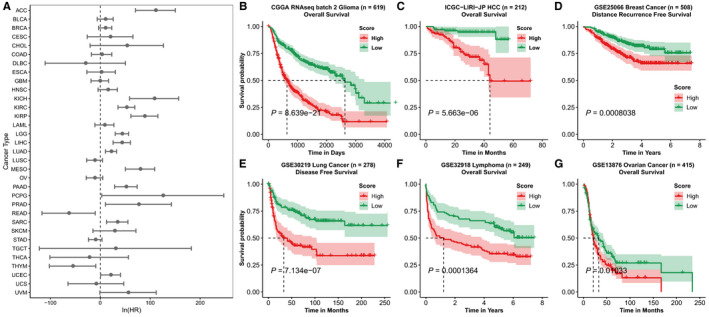
Progenitorness score predicts the survival of cancer patients. A, Analysis between progenitorness score and survival of different cancer types in TCGA, ln(hazard ratio) and 95% confidence interval (95% CI) of progenitorness score using Cox proportional hazards regression models were shown. 95% CI that does not include zero is considered significant. (B‐G) Kaplan‐Meier curve of survival in different tumour gene expression datasets. Group was separated by the median value of progenitorness scores. Differences between two curves were estimated by log‐rank test. B, CGGA RNAseq batch 2. C, Liver Cancer – RIKEN, Japan Project from International Cancer Genome Consortium, processed by HCCDB. D, GSE25066 breast cancer. E, GSE30219 lung cancer. F, GSE32918 lymphoma. G, GSE13876 ovarian cancer

Survival analysis found that higher progenitorness score indicates shorter survival time in various cancers in TCGA (Figure 2A; Figure S3). Meanwhile, 16 datasets of 7 types of cancers with survival information were collected from CGGA, HCCDB and GEO datasets. K‐M curves showed that patients with higher progenitorness scores had shorter overall/recurrent‐free/disease‐free survival time (Figure 2B‐G; Figure S4). Cox regression also confirmed that progenitorness score was an effective prognostic risk factor in survival (Tables 1 and 2). After being adjusted with age, gender, histology and WHO grade, progenitorness score was demonstrated to be an independent risk factor for glioma (Table 1).

**Table 1:** The predictive ability on survival time of progenitorness score adjusted using age, gender, WHO grade and histology

Datasets	Unadjusted			Adjusted		
	n	Hazard Ratio (95% CI)	p	n	Hazard Ratio (95% CI)	p
CGGA Microarray	298	43.6 (11.9‐160)	1.23 × 10^−8^	293	3.11 (0.766‐12.7)	0.112
CGGA RNAseq batch 1	311	3.85 × 10^9 ^(3.67 × 10^7^‐4.04 × 10^11^)	1.47 × 10^−20^	307	31 800 (126‐8.02 × 10^6^)	2.38 × 10^−4^
CGGA RNAseq batch 2	619	8.57 × 10^7^ (3.08 × 10^6^‐2.38 × 10^9^)	5.04 × 10^−27^	618	1.63 × 10^5^ (3420‐7.77 × 10^6^)	1.15 × 10^−9^
TCGA GBM + LGG	695	4.04 × 10^20^ (1.5 × 10^17^‐1.09 × 10^24^)	5.48 × 10^−32^	634	2.14 × 10^8^ (2610‐1.75 × 10^13^)	8.92 × 10^−4^
GSE4412‐GPL96	85	1.19 × 10^5^ (23.2‐6.14 × 10^8^)	0.00733	85	391 (0.0333‐4.59 × 10^6^)	0.212

Note

Hazard ratio (HR) and 95% confidence interval (95% CI) of progenitorness score using univariate and multivariate Cox proportional hazards regression models for gliomas were shown. HR with 95% CI that does not include one is considered significant.

**Table 2:** The predictive ability on survival time of progenitorness score in several types of cancers

Datasets	Cancer type	Survival type	n	HR (95% CI)	*P*
HCCDB ICGC‐LIRI‐JP	Liver	OS	212	3.07 × 10^14^ (2.61 × 10^8^‐3.59 × 10^20^)	2.89 × 10^−6^
HCCDB TCGA‐HCC	Liver	OS	351	9.35 × 10^7^ (77 500‐1.13 × 10^11^)	3.99 × 10^−7^
GSE25066	Breast	DRFS	508	3600 (9.62‐1.35 × 10^6^)	0.00675
GSE32603	Breast	RFS	248	119 (10.1‐1410)	1.48 × 10^−4^
GSE30219	Lung	OS	293	7.95 × 10^6^ (75 300‐8.38 × 10^8^)	2.33 × 10^−11^
GSE30219	Lung	DFS	278	2.83 × 10^8^ (4.11 × 10^5^‐1.95 × 10^11^)	5.32 × 10^−9^
GSE37745	Lung	OS	196	1510 (2.82‐8.06 × 10^5^)	0.0224
GSE41271	Lung	OS	274	2.42 × 10^7^ (398‐1.47 × 10^12^)	0.00249
GSE41271	Lung	RFS	274	1.09 × 10^6^ (18.5‐6.38 × 10^10^)	0.0131
GSE42127	Lung	OS	176	5.59 × 10^7^ (5.52‐5.65 × 10^14^)	0.0302
GSE50081	Lung	OS	181	4430 (1.52‐1.29 × 10^7^)	0.0391
GSE32918	Lymph	OS	249	2.20 × 10^5^ (87‐5.58 × 10^8^)	0.00209
GSE13876	Ovary	OS	415	82 (1.93‐3490)	0.0213
GSE62452	Pancreas	OS	65	2.39 × 10^7^ (361‐1.59 × 10^12^)	0.00271

Abbreviations: DFS, disease‐free survival; DRFS, distance recurrence‐free survival; OS, overall survival; RFS, recurrence‐free survival.

Hazard ratio (HR) and 95% confidence interval (95% CI) of progenitorness score using univariate Cox proportional hazards regression models were shown. HR with 95% CI that does not include one is considered significant.

### 3.3 Progenitorness score indicates tumour grades

**Figure 3:**
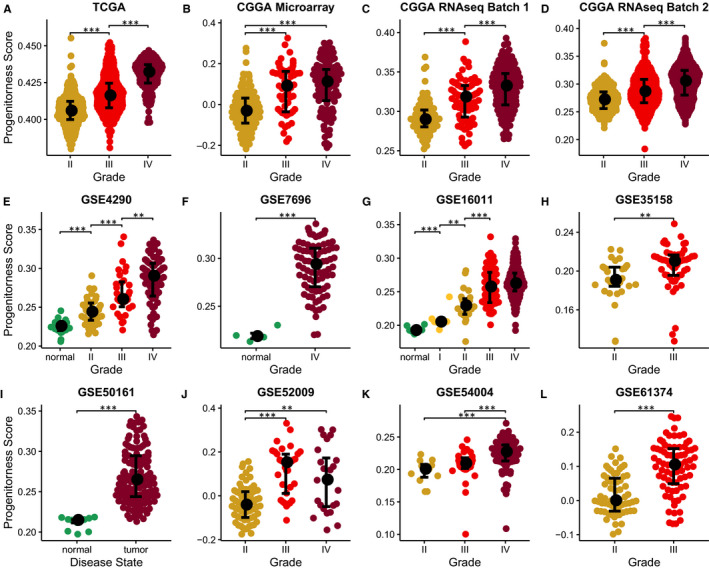
Progenitorness score indicates glioma grades. Distribution of progenitorness scores in different grade of gliomas or normal brain tissues. Significances of difference between two groups were analysed by two‐side Wilcoxon rank‐sum test. *P < 0.05, ** *P* < 0.01, ***P < 0.001. A, TCGA lower grade glioma (LGG) and glioblastoma multiforme (GBM). B, CGGA Microarray. C, CGGA RNAseq batch 1. D, CGGA RNAseq batch 2. E, GSE4290. F, GSE7696. G, GSE16011. H, GSE35158. I, GSE50161. J, GSE52009. K, GSE54004. L, GSE61374

We first studied the relationship between progenitorness score and WHO grade of glioma and found that cancers with higher grade had significantly higher progenitorness score in all 12 datasets from TCGA, CGGA and GEO datasets (Figure 3). It is worth mentioning that progenitorness score also shows the ability to distinguish glioma from normal brain tissues (Figure 3E‐G,I; Figure S5). It needs to be noted that in other type of cancers, progenitorness score has less impact on the assessment of tumour stage (Figure S6).

### 3.4 Progenitorness score guides cancer therapy

**Figure 4:**
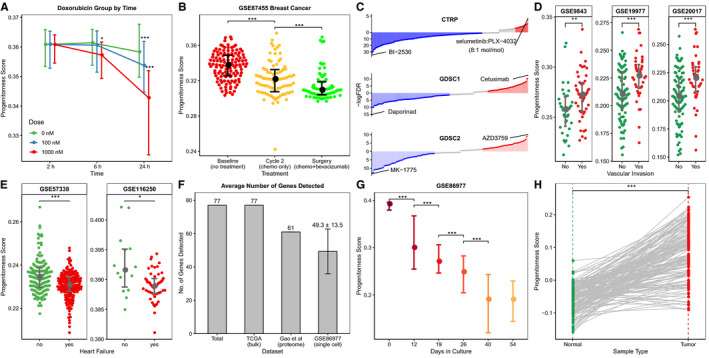
Progenitorness score guides cancer therapy and shows a wider application scenario. A, Variation of progenitorness scores in NCI‐60 cell lines treated with Doxorubicin in different drug concentration and treatment time. B, Distribution of progenitorness scores in breast cancer patients with different treatments. C, Drugs correlated with progenitorness scores in CTRP and two versions of GDSC. D, Progenitorness scores of HCC patients with/without vascular invasion. E, Distribution of progenitorness scores in heart with/without heart failure. F, Number (mean ± sd) of genes detected (read count > 0) in different datasets. G, Distribution of progenitorness scores in human embryonic stem cells of different culture days. (A, B, D, E, G) Two‐side Wilcoxon rank‐sum test, *P < 0.05, **P < 0.01, ***P < 0.001. H, Variation of progenitorness scores in an HCC‐normal paired proteome data. Wilcoxon signed‐rank test, ***P < 0.001

From the GEO gene expression data of tumour cell lines treated with various anticancer drugs (GSE116436), we observed a significant decrease of progenitorness score with the increase of drug concentration and the extension of treatment time (Figure 4A; Figure S7; Figure S8). In addition to cell trials, cancer samples of breast cancer patients also showed a decrease in progenitorness score after treatment (Figure 4B). To further investigate the relationship between progenitorness score and drug response, we collected the sensitivity to anticancer drugs of cell lines from the Cancer Therapeutics Response Portal (CTRP) and the Genomics of Drug Sensitivity in Cancer (GDSC). Area under concentration‐viability curve^24^ (AUC, the higher, the more resistant) is used to calculate the Spearman's correlation with progenitorness score. We found that the sensitivity of most drugs was significantly correlated with progenitorness score (FDR < 0.05) (Figure 4C, File S2). For example, the AUC of Cetuximab and AZD3759, which both are epidermal growth factor receptor (EGFR) inhibitors,^25^, ^26^ were found to have the most significant positive correlation with progenitorness score in two versions of GDSCs separately, which suggest a resistance to EGFR inhibitors for tumours with high progenitorness scores. The above results suggest that progenitorness score could be used to guide drug selection, either predict the efficacy before treatment or evaluate the efficacy after treatment.

### 3.5 Progenitorness score shows a wider application scenario

Furthermore, progenitorness score showed a significant predictive effect on vascular invasion of HCC (Figure 4D). It is worth mentioning that, in addition to cancer, progenitorness score also showed reasonable results in cardiovascular diseases. For example, we found that the diseased hearts have lower progenitorness scores than the normal hearts (Figure 4E).

Besides microarray and bulk RNA sequencing, single‐cell RNA sequencing (scRNAseq) data and proteome data have been also accumulated rapidly in recent years. Yao et al^27^ studied the differentiation from human embryonic stem cells (hESCs) to early forebrain and mid/hindbrain cells using scRNAseq technique. We downloaded the scRNAseq data (GSE86977) of hESCs for different culture days during differentiation from GEO datasets and then calculated their progenitorness scores. Although fewer genes were detected in scRNAseq data (Figure 4F), it showed ideal and reasonable evaluation results: progenitorness score decreased significantly with time (Figure 4G). Meanwhile, we asked whether progenitorness score works well on protein expression data. For doing so, we downloaded the proteome data of an HCC‐normal paired study by Gao et al^19^ The result showed that progenitorness score showed significant ability to classify cancer samples from normal tissues (Figures 1C and 4H).

## 4 DISCUSSION

From the atavism of cancer, we collected 77 essential genes appeared at the age of cellular organisms and eukaryote and proposed the progenitorness score as a biomarker for multi usages of multi cancers. Biomarkers are often difficult to popularize due to different batches, different platforms (microarray, bulk RNAseq, scRNAseq, proteome), different types of cancers and tissues, different processing methods of original data.^3^ But the progenitorness score was validated by dozens of datasets from TCGA, GEO, CGGA and HCCDB, etc Although fewer genes (proteins) were detected, and mRNA can only explain about 40% of the variability of protein,^28^ progenitorness score is robust in scRNAseq and proteome data.

It has been reported that older genes show greater necessity,^29^ we also found this phenomenon (Figure S9A). In addition, gene expression at each stage of embryonic development also showed a correlation with gene age.^30^ To further explore the function of gene set that we collected, we performed GO enrichment analysis. The results showed that the genes were enriched on functions about protein degradation, DNA replication and cell cycle (Figure S9B). These results may be due to a common characteristic of ancestral organisms, embryos and cancers: rapid proliferation, which partly explains why progenitorness score is effective in a wide variety of tumours. However, it needs to be pointed out that our progenitorness gene set has no intersection with stemness gene set in the Kyoto Encyclopedia of Genes and Genomes^31^ (KEGG, pathway id: hsa04550) and PathCards,^32^ which reveals a new perspective on cancer progression.

We also tried to reduce the gene set and optimize it for various tissue types separately. RNA sequencing data for different tissues from the Genotype‐Tissue Expression (GTEx)^33^ database have been downloaded and the specificities of each progenitor gene in various tissues were calculated. We then applied the specific gene sets to TCGA cancers of the corresponding tissues. However, the diagnostic and prognostic abilities of these gene sets were not increased, comparing with the original 77 genes (data not shown).

An interesting phenomenon is that in rectum adenocarcinoma (READ) of TCGA, progenitorness score shows a negative correlation with survival (HR = 3.00 × 10^−28^, *P* = 0.0199). At the same time, stomach adenocarcinoma (STAD) showed the same but not significant trend (HR = 4.44 × 10^−5^, *P* = 0.147). Similar results were observed in the analysis of colorectal and gastric cancer data from GEO datasets (Figure S10), which were probably not by accident. The reason is worth further validating and exploring.

In summary, to explore the atavism of cancer, we have been suggested that ancient genes could serve as a biomarker of cancer. As a result, 77 progenitor genes were collected and applied to calculate progenitorness score. After the verification of dozens of cancer gene expression data, progenitorness score has been found to be used for the diagnosis, prognosis, grading, medication guidance and other purposes of a variety of cancers. Furthermore, we revealed the possible role of ancient genes in the development of cancer and showed an atavistic landscape of cancer.

## CONFLICT OF INTEREST

The authors declare no competing interests.

## AUTHOR CONTRIBUTIONS

QC conceived the project. XJ performed the analysis and conducted the experiments. XJ and QC wrote the manuscript. All authors read and approved the final manuscript.

## Supporting information

Fig S1‐S10

Table S1

Table S2

## Data Availability

The data that support the findings of this study are openly available in GDC data portal at https://portal.gdc.cancer.gov, the Chinese Glioma Genome Atlas at http://www.cgga.org.cn, the Integrative Molecular Database of Hepatocellular Carcinoma at http://lifeome.net/database/hccdb[16], GEO database at https://www.ncbi.nlm.nih.gov/gds, Cancer Cell Line Encyclopedia at https://portals.broadinstitute.org/ccle[20], Cancer Therapeutics Response Portal at http://portals.broadinstitute.org/ctrp[21], Genomics of Drug Sensitivity in Cancer at https://www.cancerrxgene.org[22], Genotype‐Tissue Expression at https://www.gtexportal.org/home[33]. The data that supports the findings of this study are available in the supplementary material of these articles.^18^, ^19^
