# Supplementary material for: Ancient genes can be served as pan‐cancer diagnostic and prognostic biomarkers
Source: J Cell Mol Med. 2020 May 5;24(12):6908–15. doi: 10.1111/jcmm.15347 (PMC7299709; doi:10.1111/jcmm.15347)
Supplement: Supplementary file 1 — Fig S1‐S10 [file JCMM-24-6908-s001.pdf]

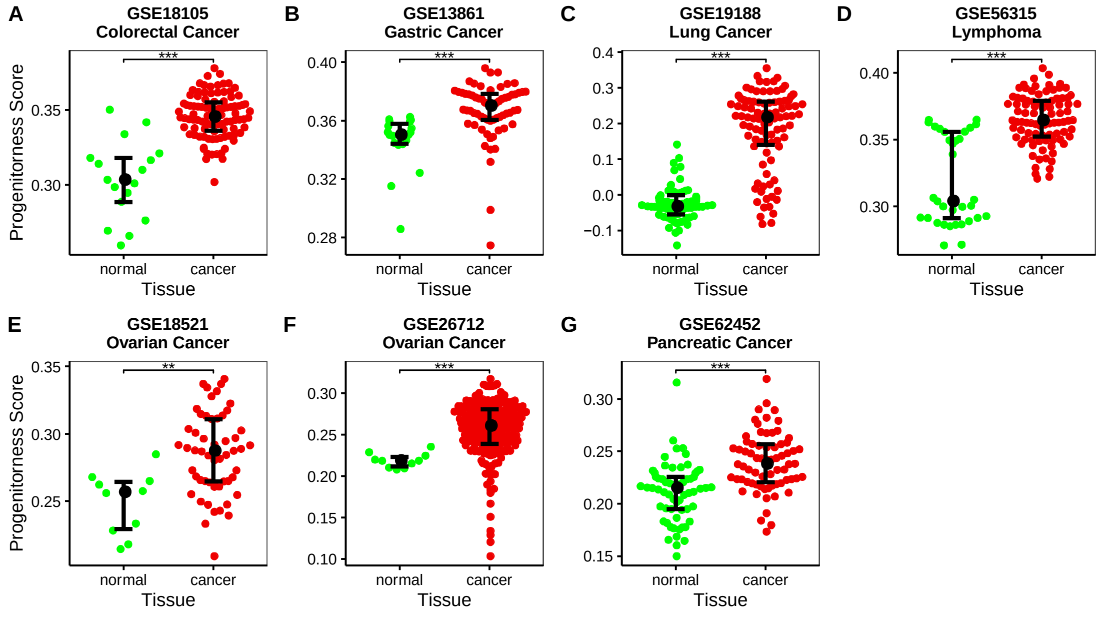

Fig. S1. Distribution of progenitorness scores in tumor and normal tissues from GEO datasets. Two-side Wilcoxon rank sum test. * p<0.05, ** p<0.01, *** p<0.001.

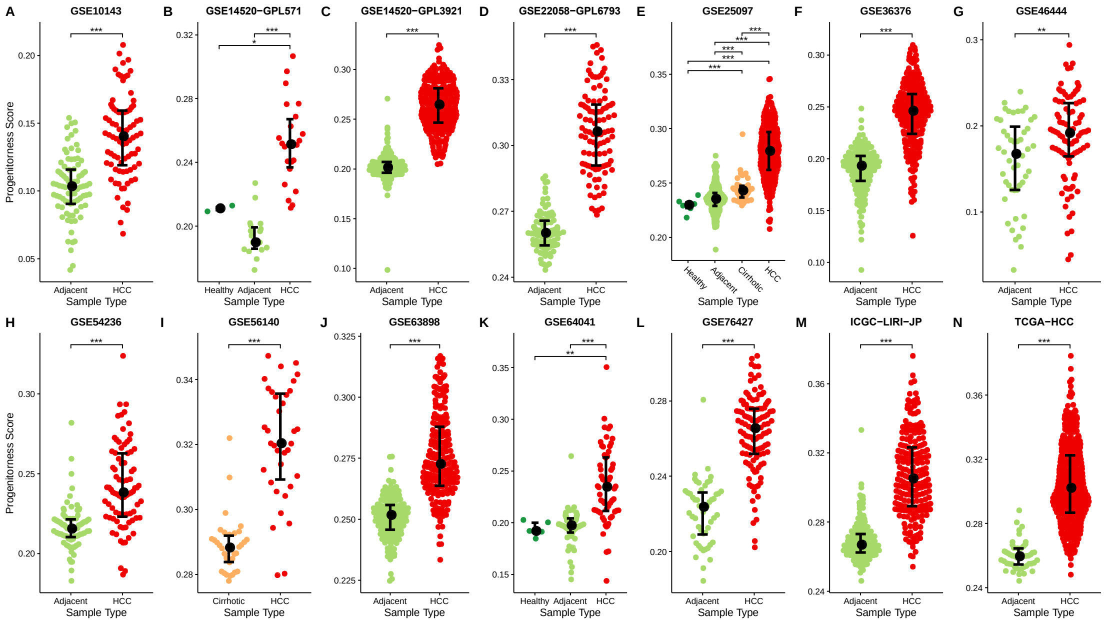

Fig. S2. Distribution of progenitorness scores in hepatocellular carcinoma (HCC) datasets. Liver samples from healthy donors and cirrhotic liver tissues are contained in some of the datasets. Two-side Wilcoxon rank sum test. * p<0.05, ** p<0.01, *** p<0.001. (a-h, j-n) Datasets processed by HCCDB. (i) GSE56140 from GEO datasets.

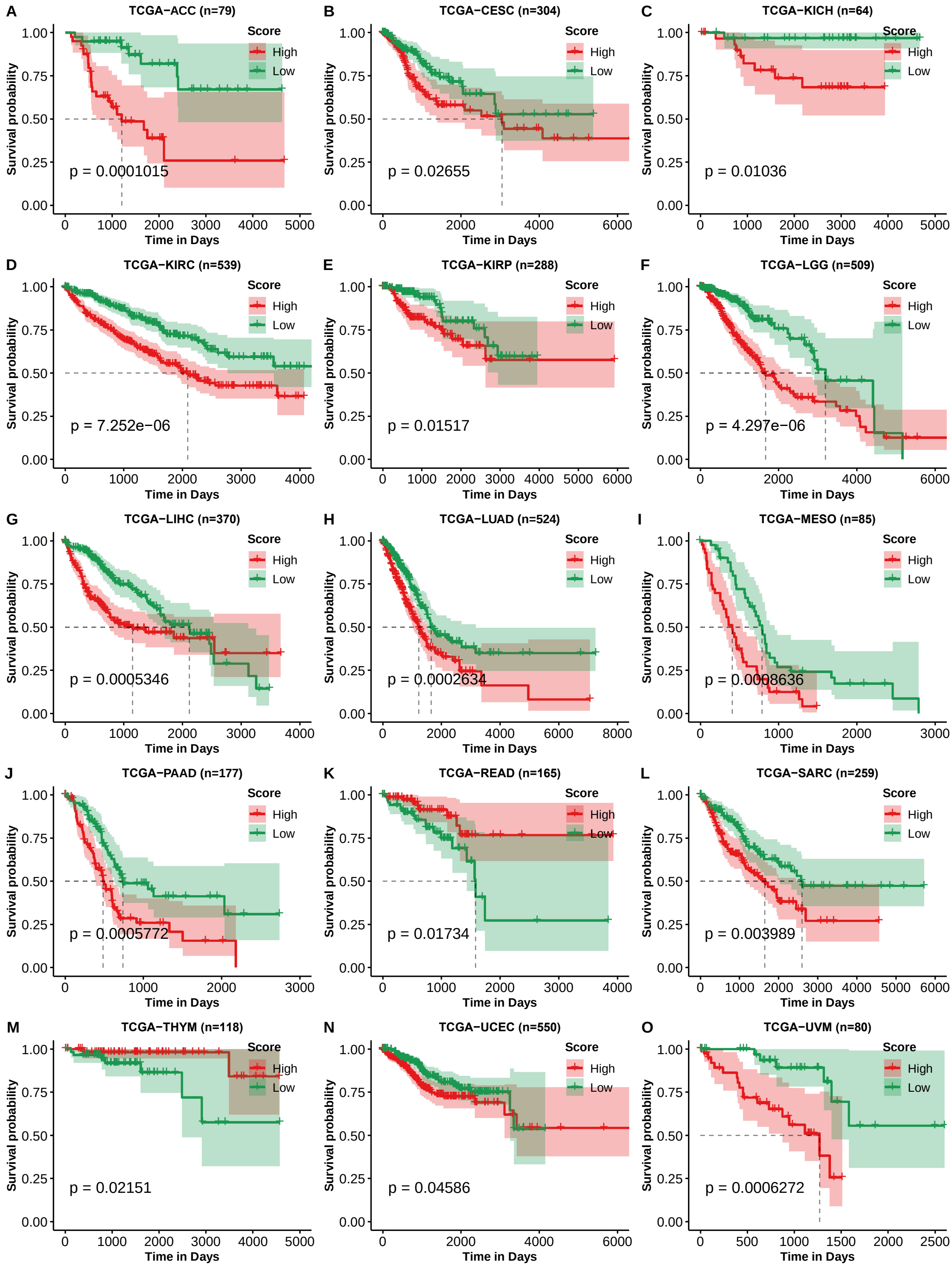

Fig. S3. Kaplan-Meier curve of overall survival in different cancer types of TCGA. Group was separated by the median value of progenitorness scores. Differences between two curves were estimated by log-rank test..

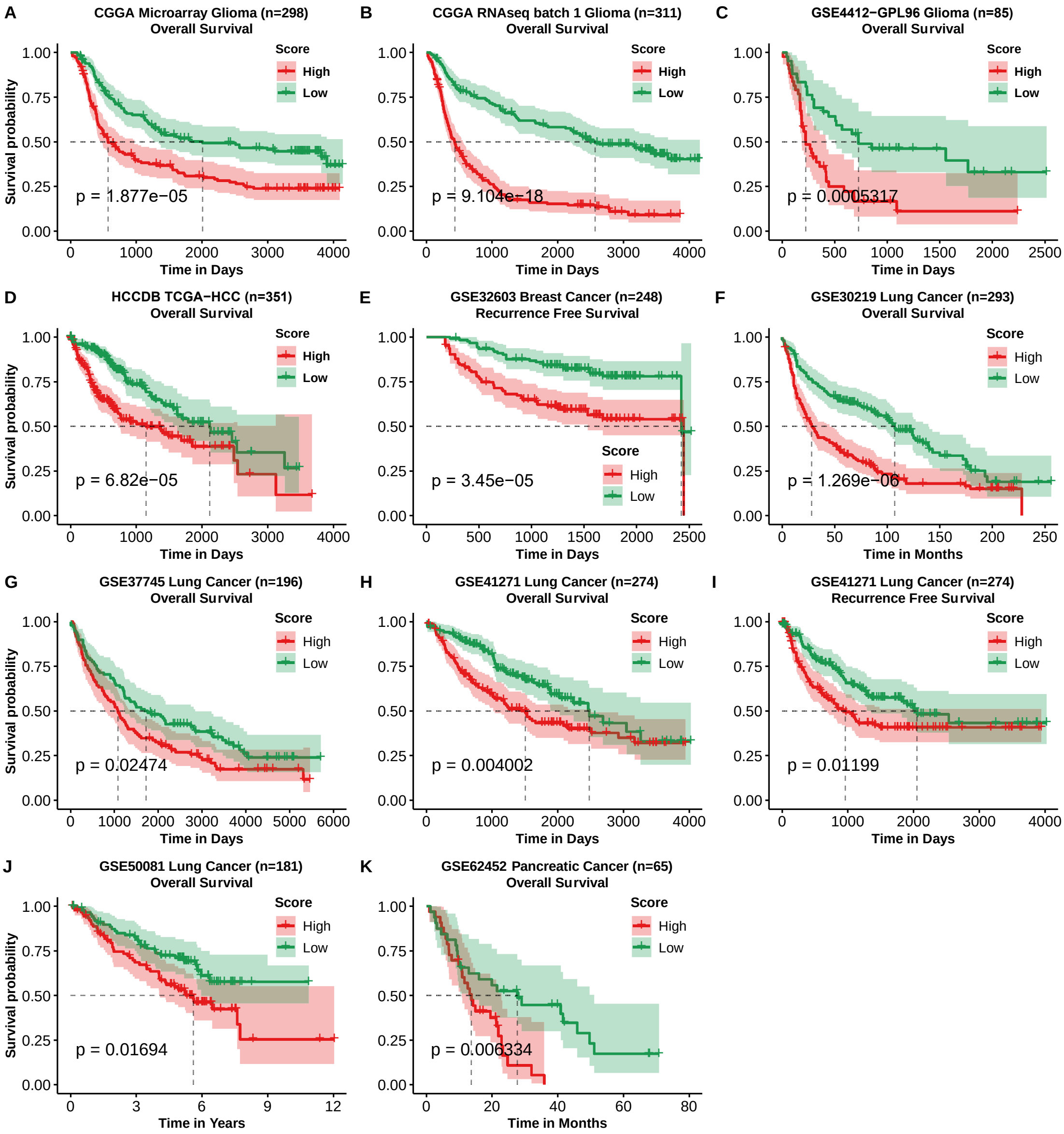

Fig. S4. Kaplan-Meier curve of overall survival or recurrence free survival in datasets of GEO. Groups were separated by the median value of progenitorness scores. Differences between two curves were estimated by log-rank test.

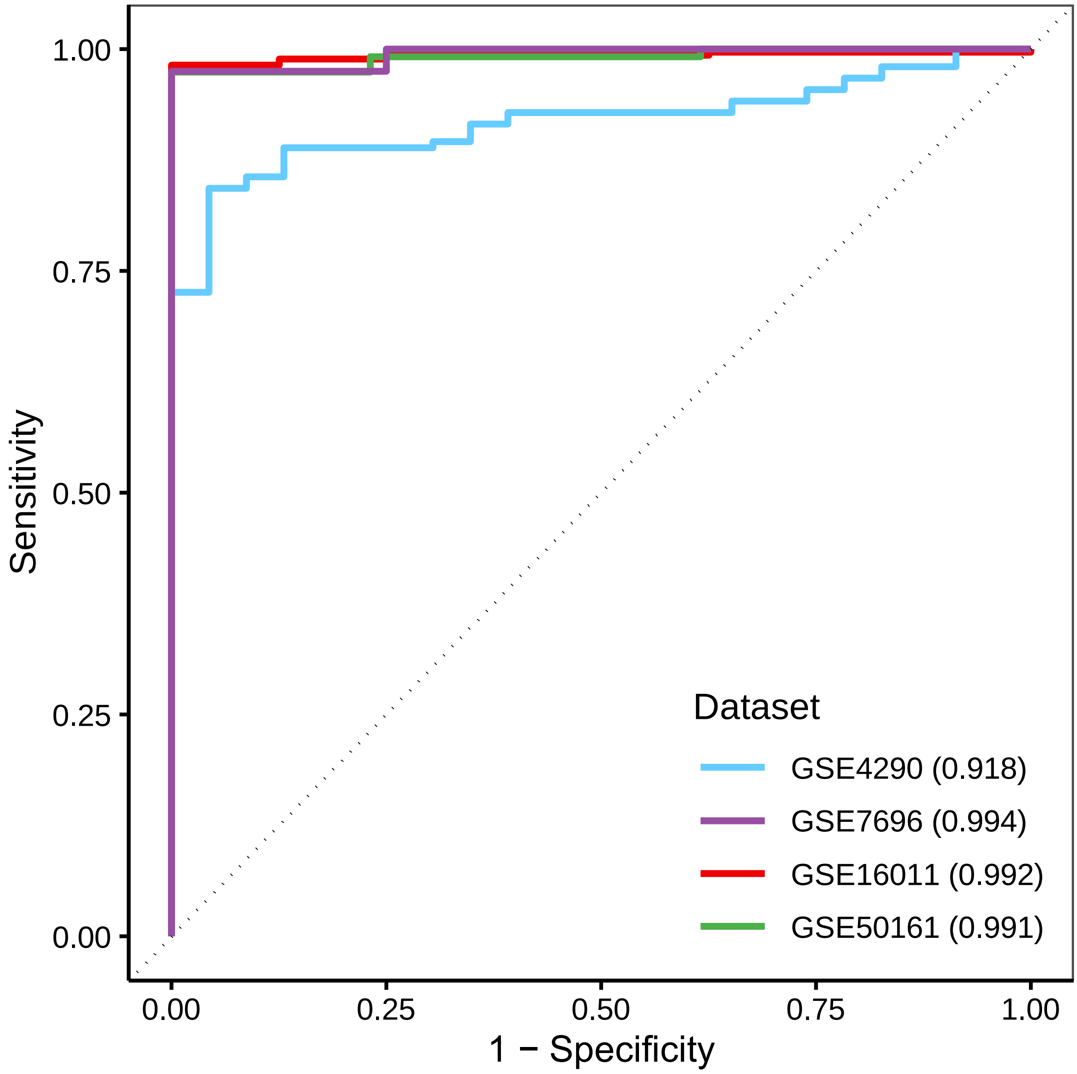

Fig. S5. Progenitorness score can discriminate gliomas from normal brain tissues. ROC curves of GSE4290 (blue), GSE7696 (purple), GSE16011 (red), GSE50161 (green) from GEO datasets. The area under ROC curves are shown in parentheses.

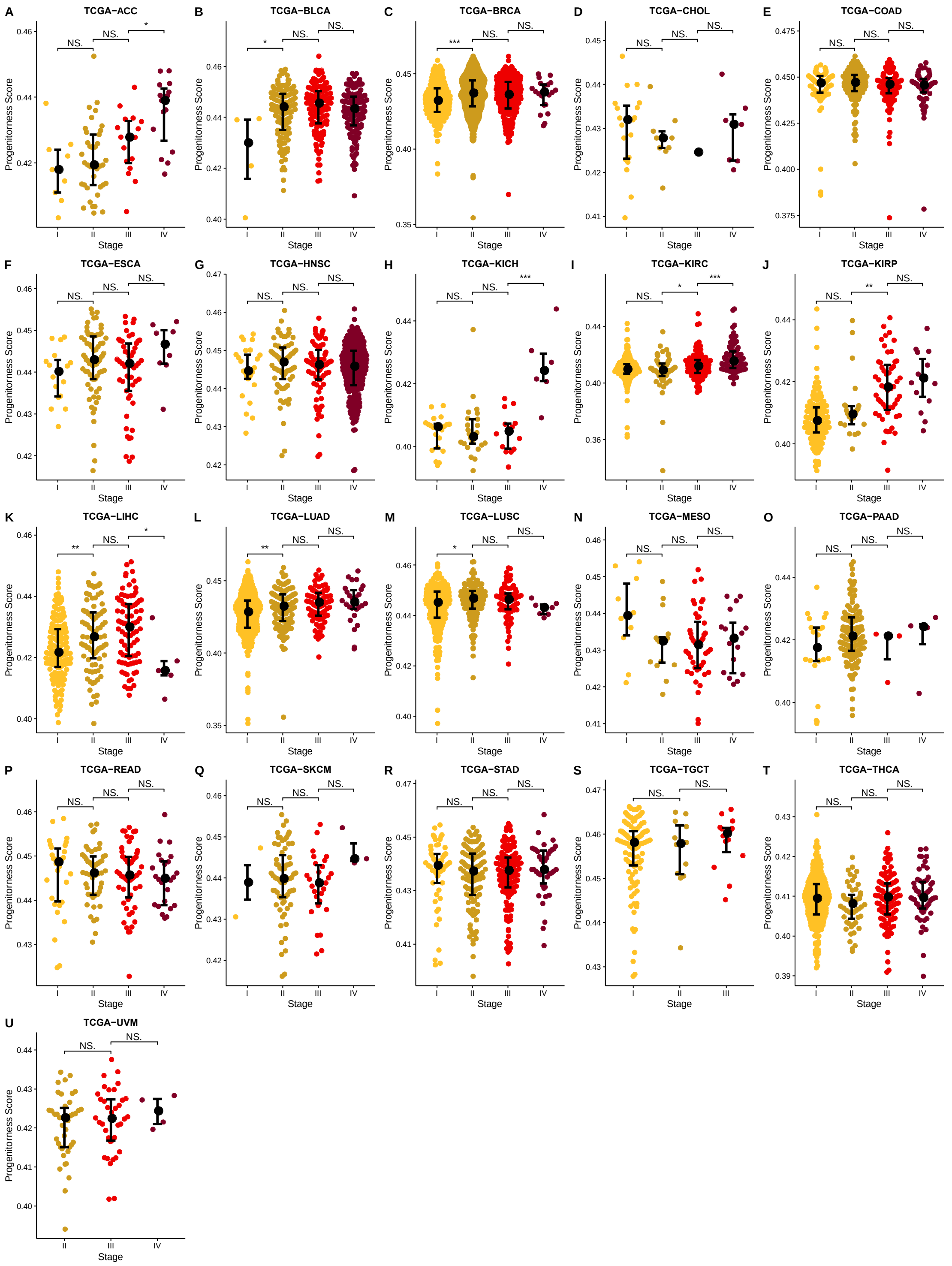

Fig. S6. Distribution of progenitorness scores in different stages of tumors from TCGA.

Two-side Wilcoxon rank sum test. NS: not significant, * p<0.05, ** p<0.01, *** p<0.001.

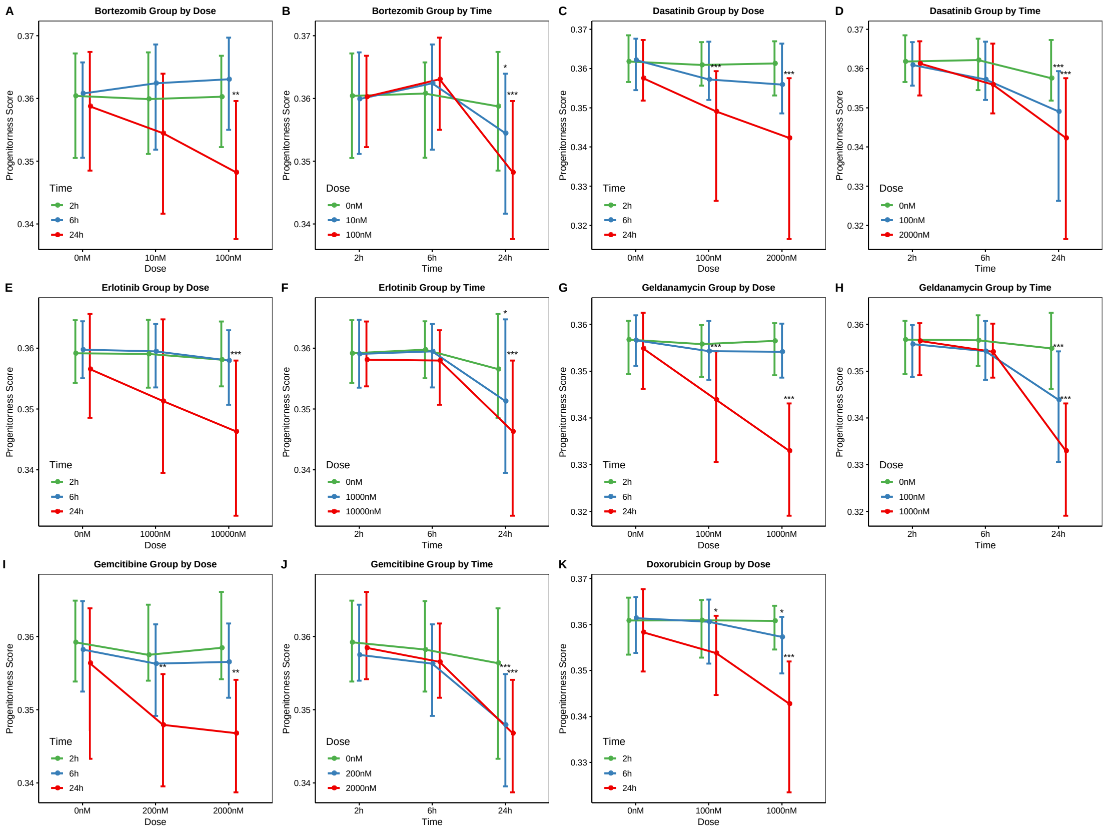

Fig. S7. Variation of progenitorness scores in NCI-60 cell lines treated with anticancer drugs. Samples were grouped by different drug concentration and treatment time. Significances of difference were calculated between one group and 2-hour-time group of the same dose or 0-nmol-dose group of the same time. Two-side Wilcoxon rank sum test. * $p<0.05$, ** $p<0.01$, *** $p<0.001$.

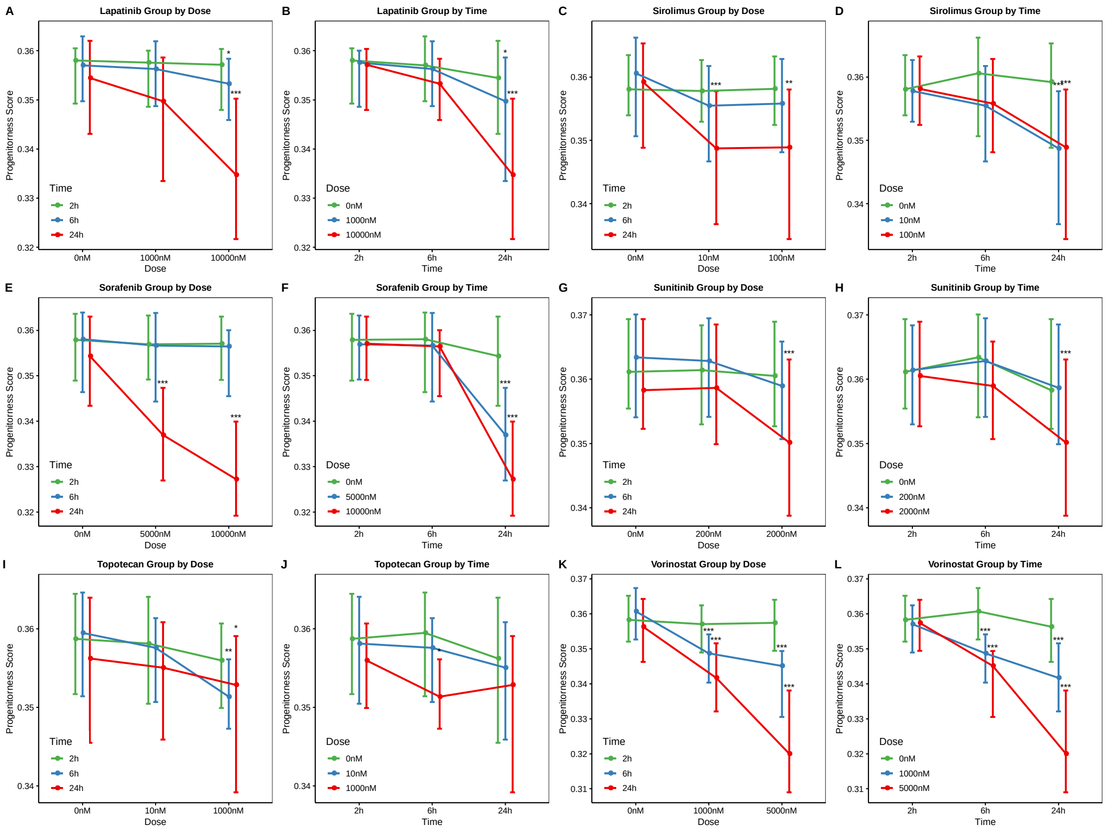

Fig. S8. Variation of progenitorness scores in NCI-60 cell lines treated with anticancer drugs. Samples were grouped by different drug concentration and treatment time. Significances of difference were calculated between one group and 2-hour-time group of the same dose or 0-nmol-dose group of the same time. Two-side Wilcoxon rank sum test. * $p<0.05$, ** $p<0.01$, *** $p<0.001$.

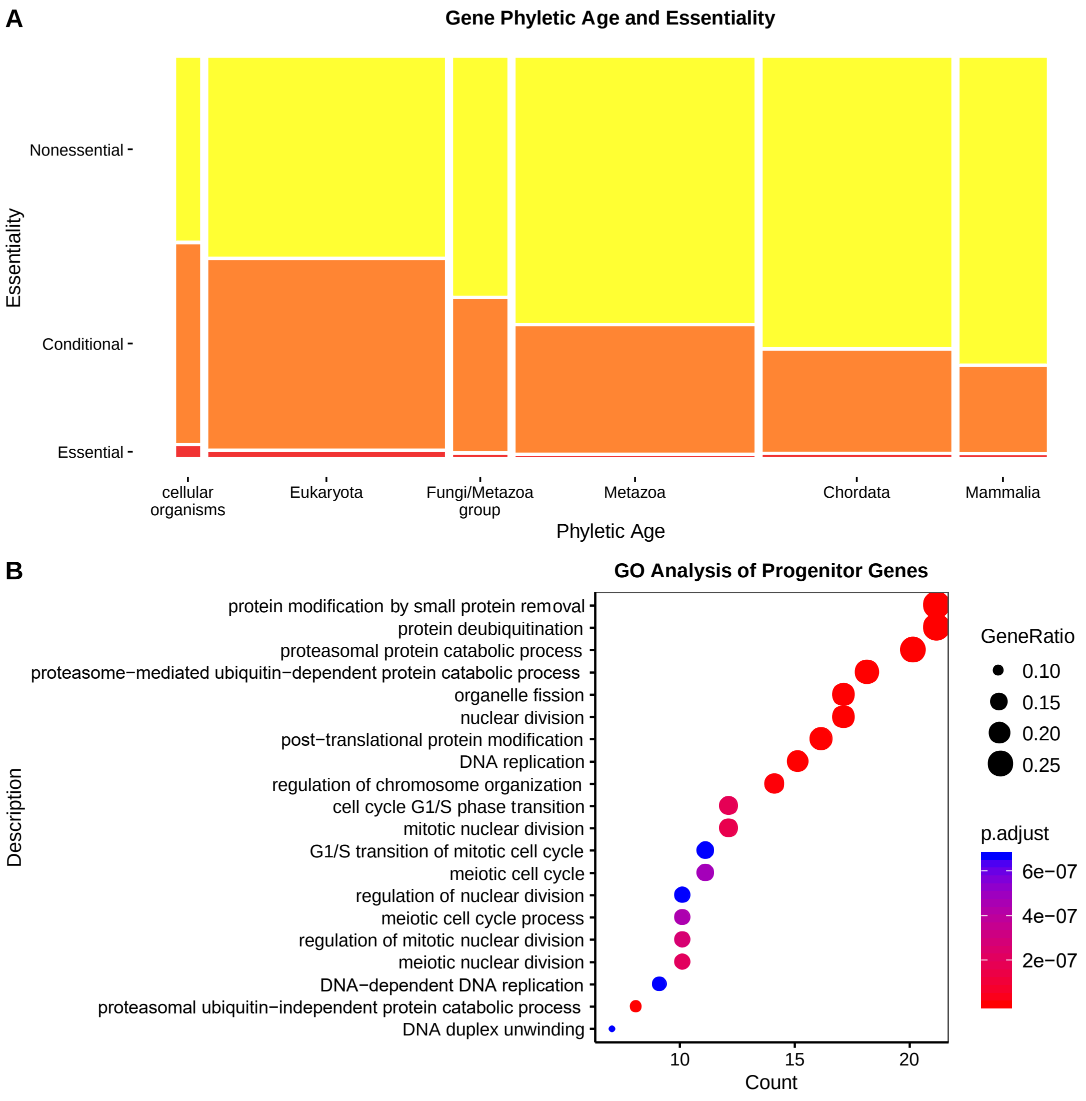

Fig. S9. The essentiality and functions of ancient genes. (a) The mosaic plot shows the correlation between gene phyletic age and essentiality. The length of the side represents the proportion of the number of certain kind of genes. (b) GO enrichment analysis of progenitor genes.

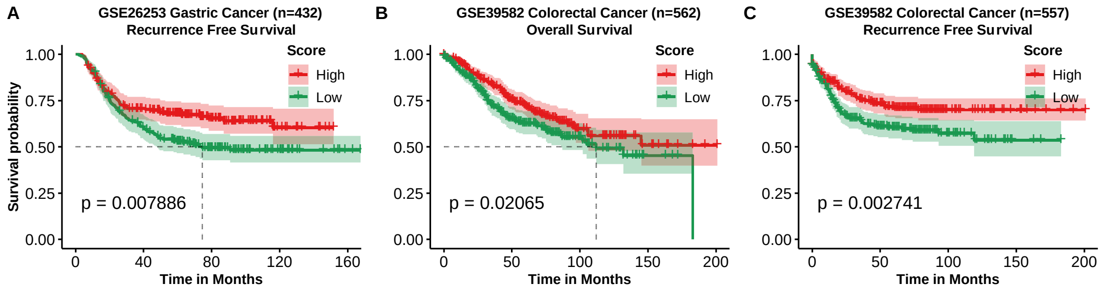

Fig. S10. Kaplan-Meier curve of overall survival or recurrence free survival in gastric and colorectal cancer datasets from GEO. Groups were separated by the median value of progenitorness scores. Differences between two curves were estimated by log-rank test. (a) GSE26253 gastric cancer recurrence free survival. (b) GSE39582 colorectal cancer overall survival. (c) GSE39582 colorectal cancer recurrence free survival.
